# Collection and extraction of water level information from a digital river camera image dataset

**DOI:** 10.1016/j.dib.2020.106338

**Published:** 2020-09-28

**Authors:** Sanita Vetra-Carvalho, Sarah L. Dance, David C. Mason, Joanne A. Waller, Elizabeth S. Cooper, Polly J. Smith, Jemima M. Tabeart

**Affiliations:** a Department of Meteorology, University of Reading, Earley Gate, Whiteknights Road, Reading RG6 6ET, United Kingdom; b Department of Mathematics and Statistics, University of Reading, Whiteknights, Pepper Lane, Reading RG6 6AX, United Kingdom; c Department of Geography and Environmental Science, University of Reading, Russell Building, Whiteknights, Pepper Lane, Reading RG6 6DR, United Kingdom; d National Centre for Earth Observation (NCEO), Meteorology Building, University of Reading, Earley Gate, Whiteknights Road, Reading RG6 6ET, United Kingdom

**Keywords:** River cameras, Water level observations, Gauge data, Flooding, Inundation

## Abstract

We present a new water level dataset extracted from images taken by four Farson Digital Ltd river cameras for a Tewkesbury, UK flood event (21st November – 5th December 2012). This data article presents the new water level data together with a description of metadata, data acquisition, and extraction methods. The water level information was extracted from the images using measured points in the field-of-view of each camera using Leica GNSS and Total Station instruments with high spatial accuracy of order of 1 cm. We use river gauge data to verify the new dataset. The new dataset has a short duration but includes the rising limb, peak discharge and falling limb of the flood event. It has potential for verifying future automatic water level extraction methods and for development of automatic flood alert methods and can provide valuable information in data assimilation systems used for improving inundation forecasts.

## Specifications Table

**Table:** 

Subject	Water Science and Technology
Specific subject area	Water level measurements in rivers
Type of data	Table Image Graph Figure Text file
How data were acquired	River camera images (from which the water-level observations were extracted) were taken by Mobotix M24 Allround outdoor web-camera with 3MP (Mega Pixel) resolution. Location metadata were measured by Leica TS 12 (TS) and Leica CS10/CS15 & GS Sensor instruments (GNSS).
Data format	Raw Analysed
Parameters for data collection	Flood event between 21st November and 5th December 2012 in the Tewkesbury, UK area.
Description of data collection	New water level data obtained from Farson Digital river cameras at four locations in the UK. Data was extracted from images manually by matching geo-referenced GNSS & TS metadata with water line points in the field-of-view of each camera.
Data source location	City/Town/Region: Evesham (Worcestershire), Tewkesbury (Gloucestershire), Diglis Lock (Worcestershire), Strensham Lock (Worcestershire). Country: United Kingdom
Data accessibility	Repository name: Mendeley Data Data identification number: DOI: 10.17632/769cyvdznp.1 Direct URL to data: http://dx.doi.org/10.17632/769cyvdznp.1

## Value of the Data

• The new water level dataset provides a proof-of-concept for the use of river cameras as a new source of information on river levels. This initial dataset can be used to verify and validate future automatic water level extraction algorithms from river cameras.
• The new water-level dataset is especially useful for case studies on short-term flood hazard predictions and longer-term flood risk management and planning. This dataset provides novel, complementary information to existing water-level data from river-gauge and satellite synthetic aperture radar (SAR) data on the Tewkesbury November 2012 flood event. In particular, the dataset provides new information on water-levels on the rising limb of the flood when the river is out-of-bank.
• This new dataset provides hourly observations during daylight. Such a temporal observation frequency means that river cameras can capture the crucial rising limb of the flood. Thus, the dataset may be used as a new source of information for use with hydrodynamic flood inundation models for calibration and data assimilation.

## 1 Data Description

**Fig. 1:**
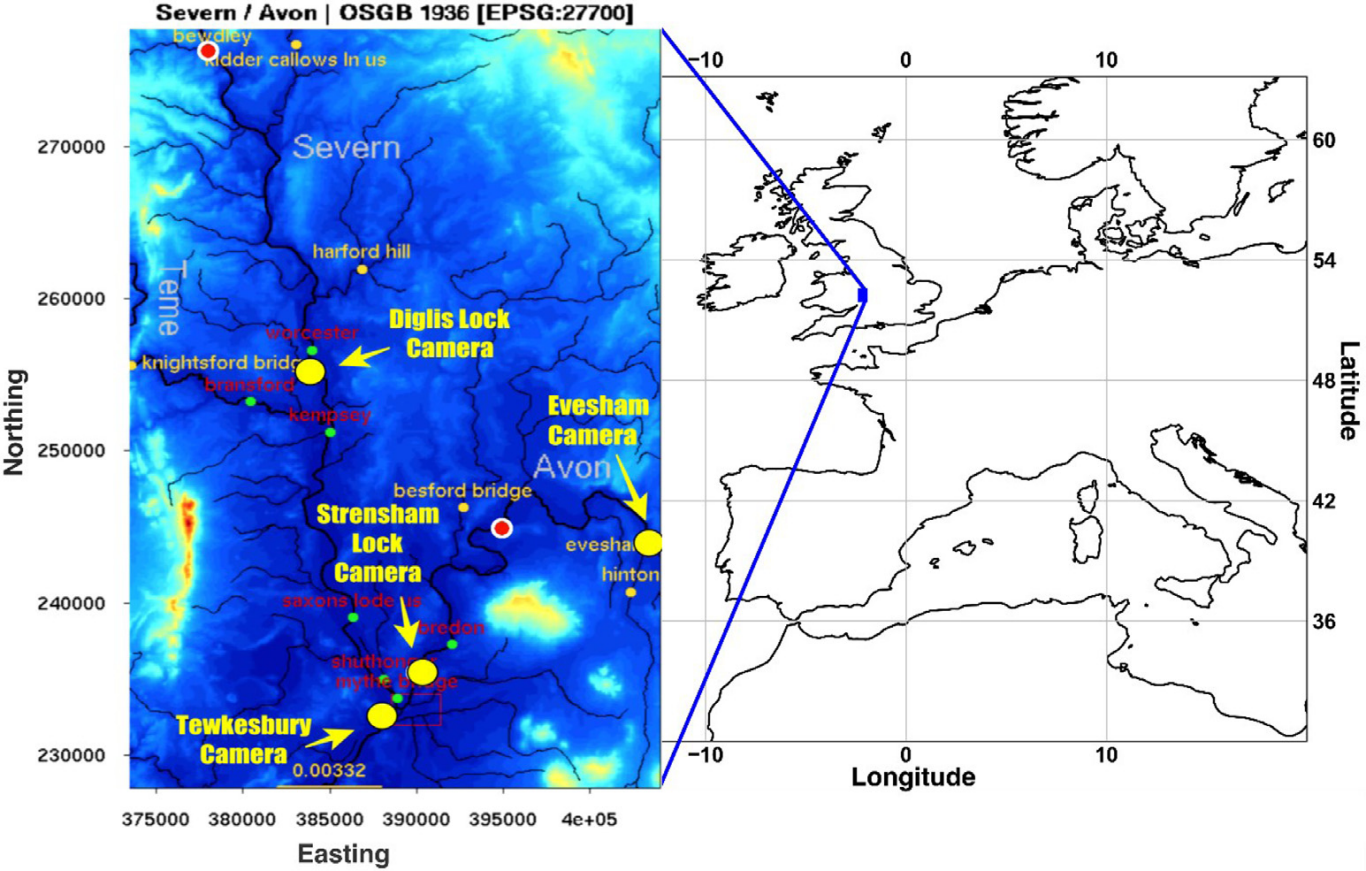
Left panel: Map showing locations of the four Farson Digital Ltd river cameras from which the new water level data was extracted: Diglis Lock (river Severn), Tewkesbury (river Avon), Strensham Lock (river Avon), and Evesham (river Avon). Original terrain image taken from [2]. The vertical and horizontal coordinates are Northing and Easting in m. The small yellow dots show nearest river flow and rainfall gauging stations. Right panel: Map of Europe showing the location of the study area. Grid lines show latitude and longitude in degrees. (For interpretation of the references to colour in this figure legend, the reader is referred to the web version of this article.)

New water level observations are derived from four river cameras: Diglis Lock, Tewkesbury, Strensham Lock, and Evesham, located on rivers Avon and Severn in the UK (see Fig. 1) and are valid for the period between 21st November and 5th December of 2012 when a major flooding event occurred around the Tewkesbury area. The Tewkesbury 2012 flood event is a well-studied and documented event, with a number of river gauges within the catchment that can be used for validation [1,2]. Rainfall is the main driver of the river level rise and fall; however, river levels can also be affected by other factors, e.g. such as the opening and closing of dams and sluices or debris collecting in culverts [3]. The interested reader can download daily cumulative rainfall data from the UK National River Flow Archive [1] for the following raingauge locations illustrated on Fig. 1: Saxons Lode, Besford Bridge, Harford Hill, Knightsford Bridge, Bredon and Evesham. In addition, there is a series of 7 synthetic aperture radar (SAR) satellite overpasses at approximately 12-h intervals during the event, but none are available on the rising limb of the flood [2].

The new river camera dataset provides hourly daytime water level information on both the River Avon and River Severn for both the rising and falling limb of the flood. Thus, the dataset can be used as an additional source of information for flood event case studies, to verify and validate future automatic water level extraction algorithms from river cameras and with hydrodynamic flood inundation models for calibration and data assimilation [2, [4], [5], [6], [7], [8], [9], [10]].

The water levels for the four river cameras over the period were manually extracted from the archived hourly daylight images using high accuracy field-of-view point measurements for each of the cameras. These were measured using GNSS and Total Station.

Details of the GNSS coordinate system at all the camera locations are provided in Table 1. Table 2 shows the 3 G network base stations used at each river camera location to ensure high accuracy GNSS measurements. The coordinate system used with Total Station measurements is given in Table 3. The 3-D coordinates for each camera location are shown in Table 4. The nearest Environment Agency (EA) river gauge stations to each of the river camera locations are given in Table 5.

**Fig. 2:**
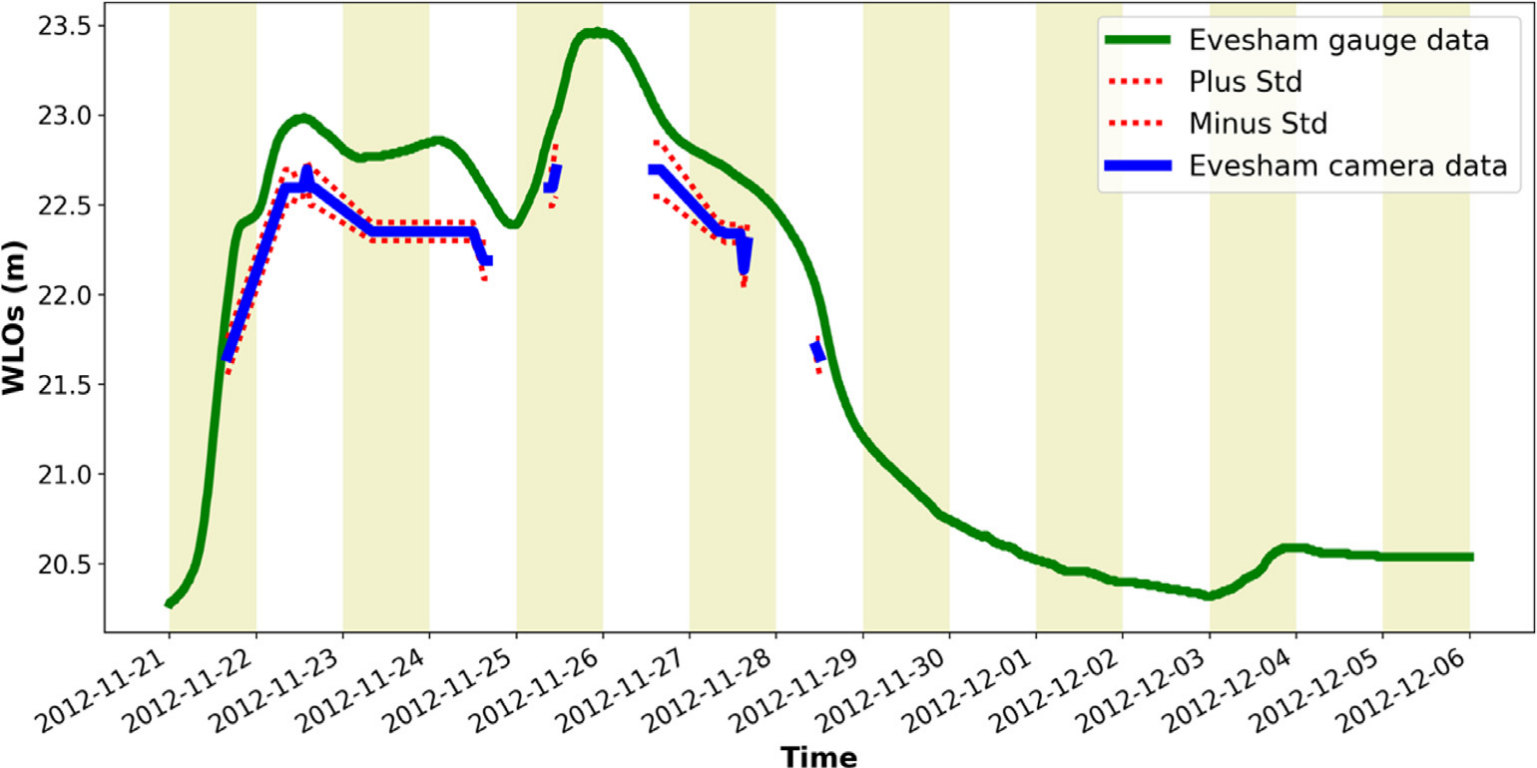
Water level information extracted from Farson Digital Ltd river camera images at Evesham. The new river-camera-derived water levels are plotted against the nearest river gauge station, Evesham, approximately 1823 m from the camera along the river.

**Fig. 3:**
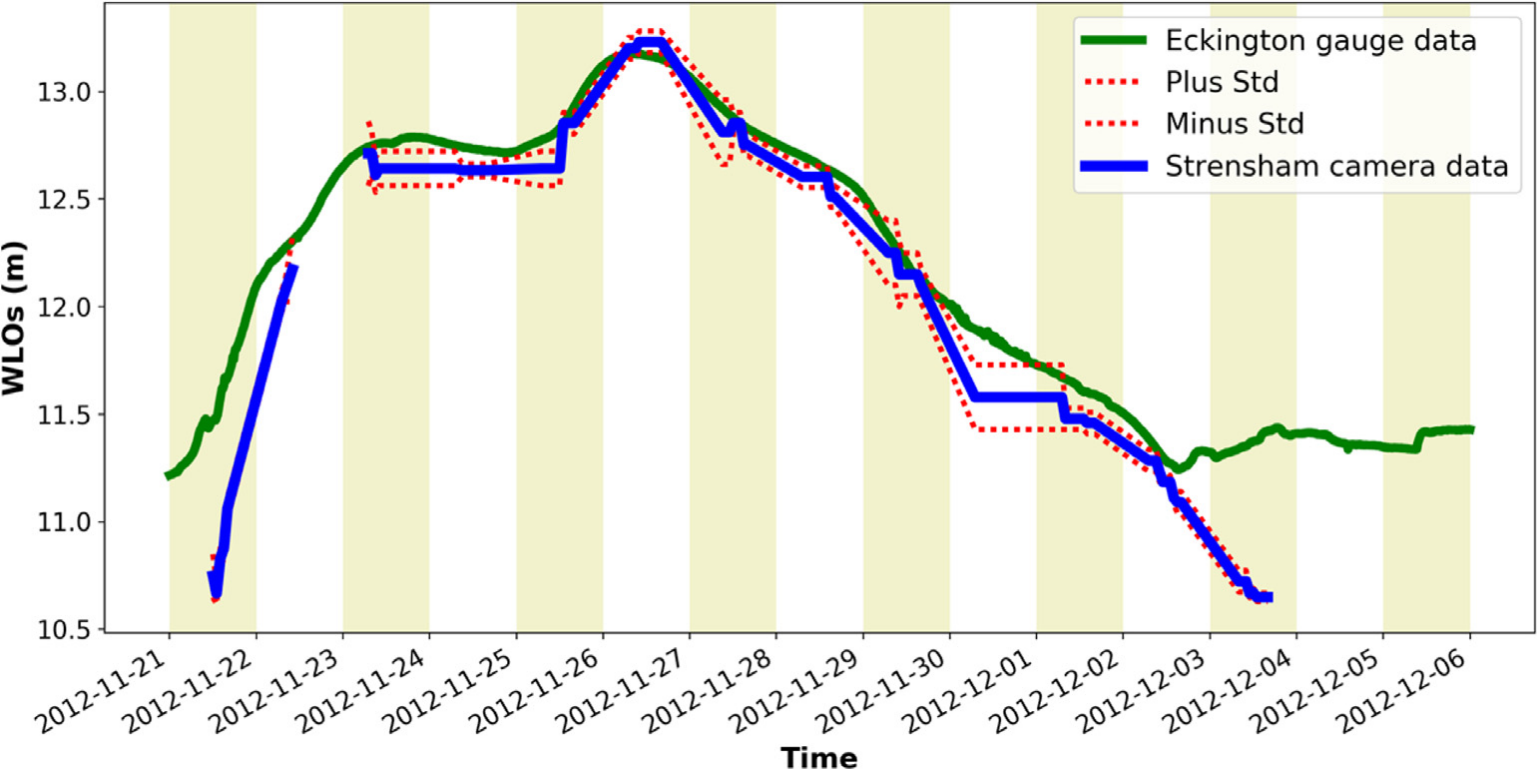
Water level information extracted from Farson Digital Ltd river camera images at Strensham. The new river-camera-derived water levels are plotted against the nearest river gauge station, Eckington Sluice, approximately 51 m from the camera along the river.

**Fig. 4:**
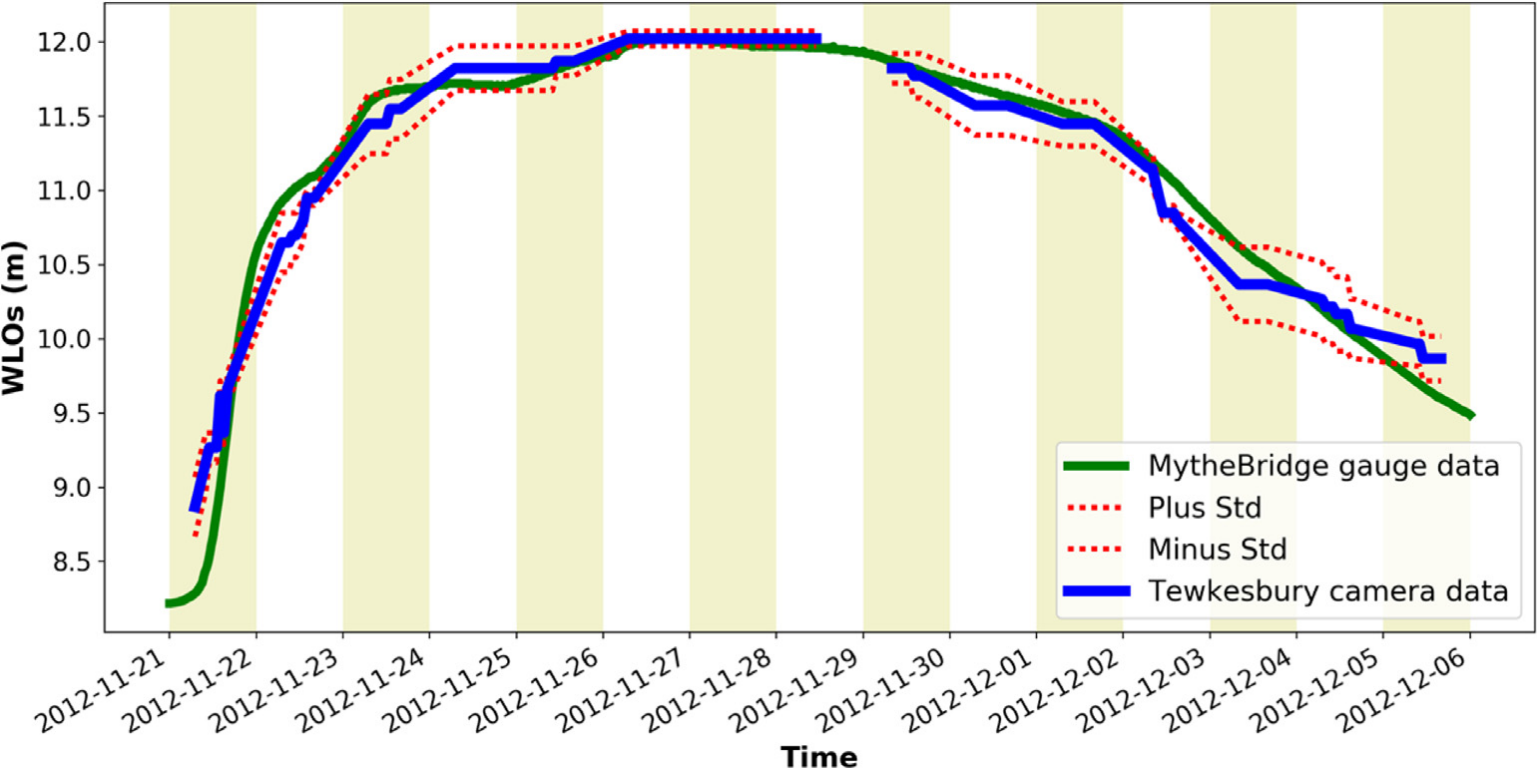
Water level information extracted from Farson Digital Ltd river camera images at Tewkesbury. The new river-camera-derived water levels are plotted against the nearest river gauge station, Mythe Bridge, approximately 1491 m from the camera along the river.

**Fig. 5:**
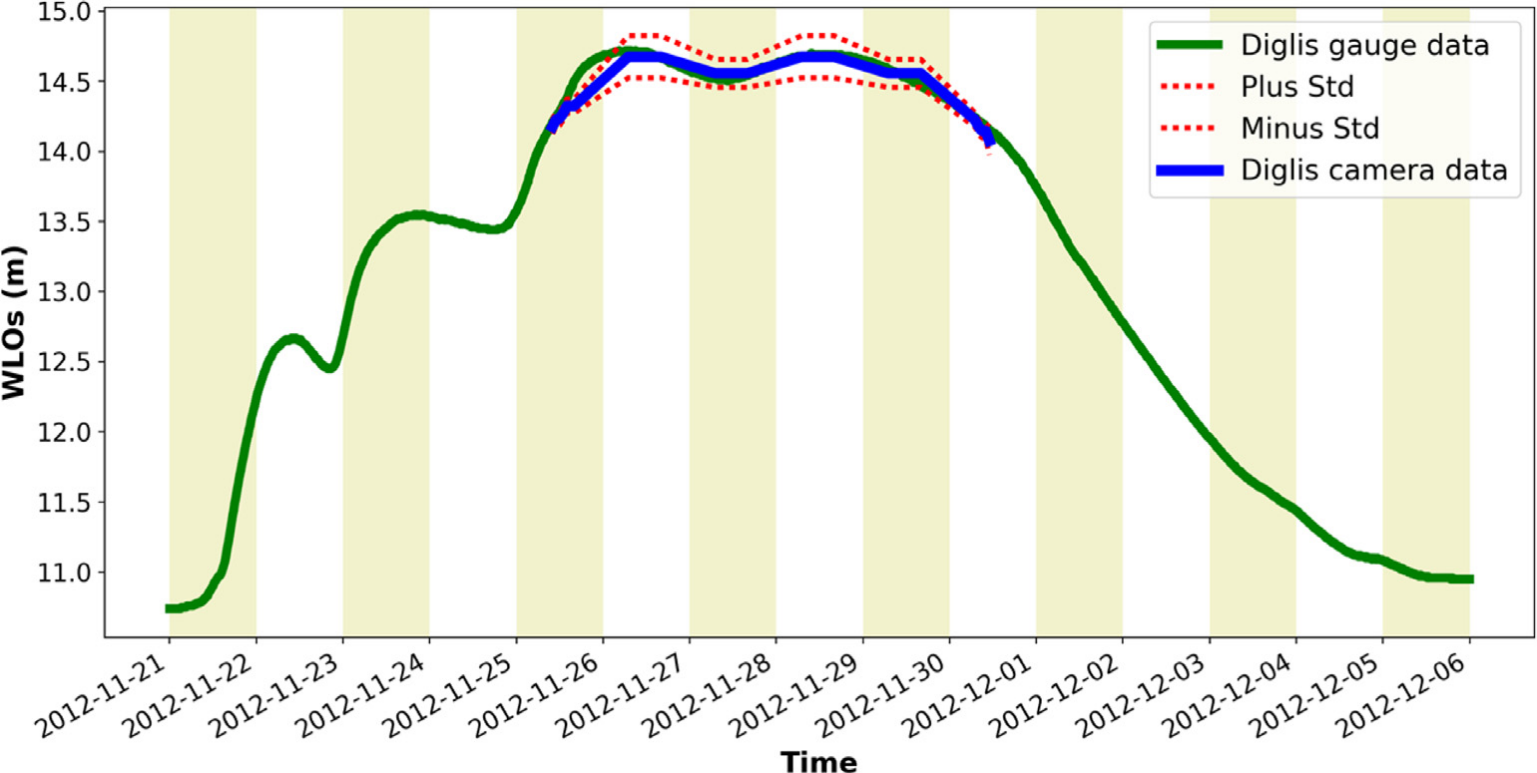
Water level information extracted from Farson Digital Ltd river camera images at Diglis Lock. The new river-camera-derived water levels are plotted against the nearest river gauge station, Diglis, approximately 274 m from the camera along the river.

**Fig. 6:**
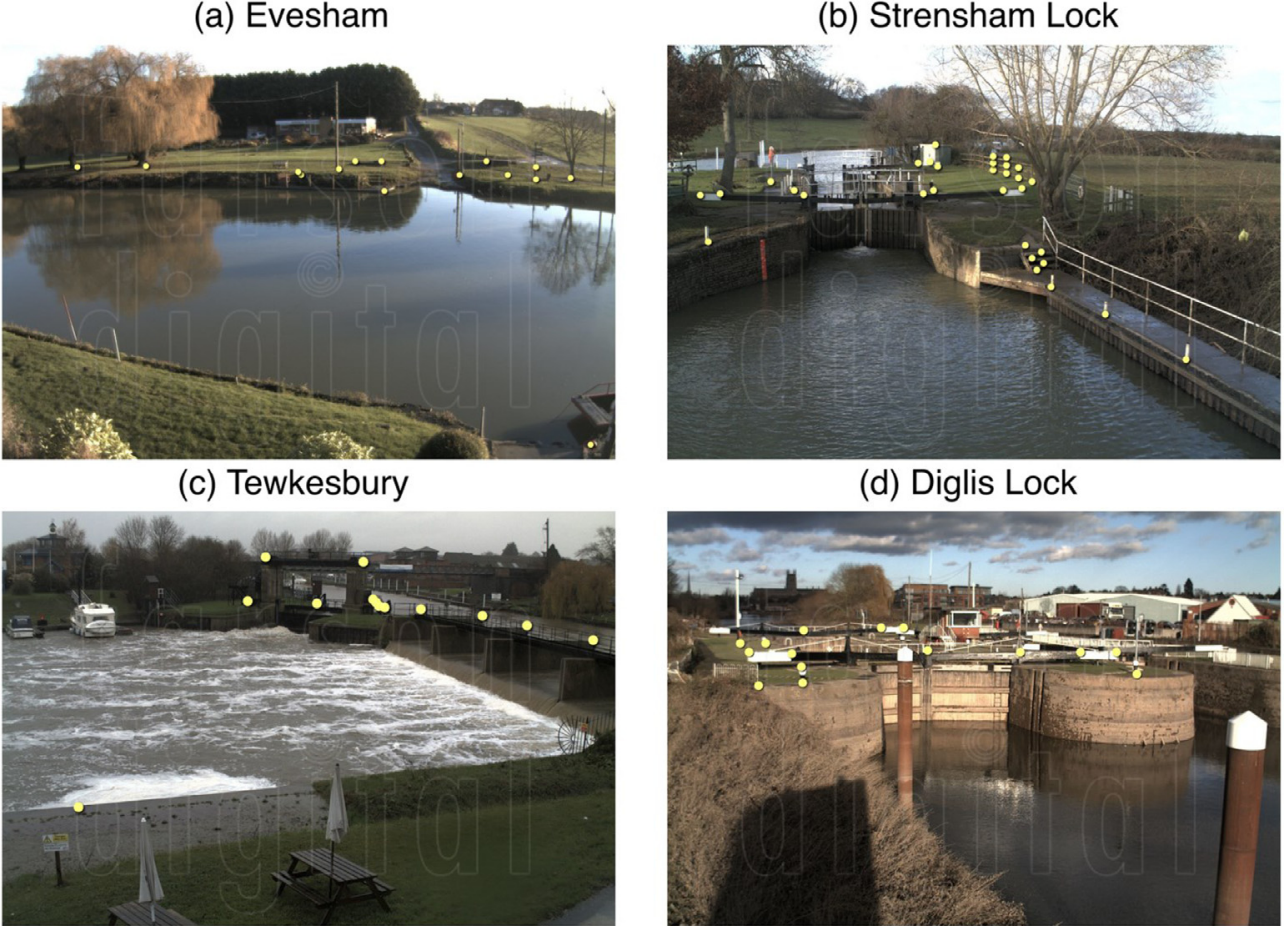
Camera views from (a) Evesham, (b) Strensham Lock, (c) Tewkesbury, (d) Diglis Lock. The yellow dots illustrate some of the measured points in the field-of-view of the cameras which were used to extract the WLOs from the image. (For interpretation of the references to colour in this figure legend, the reader is referred to the web version of this article.)

**Fig. 7:**
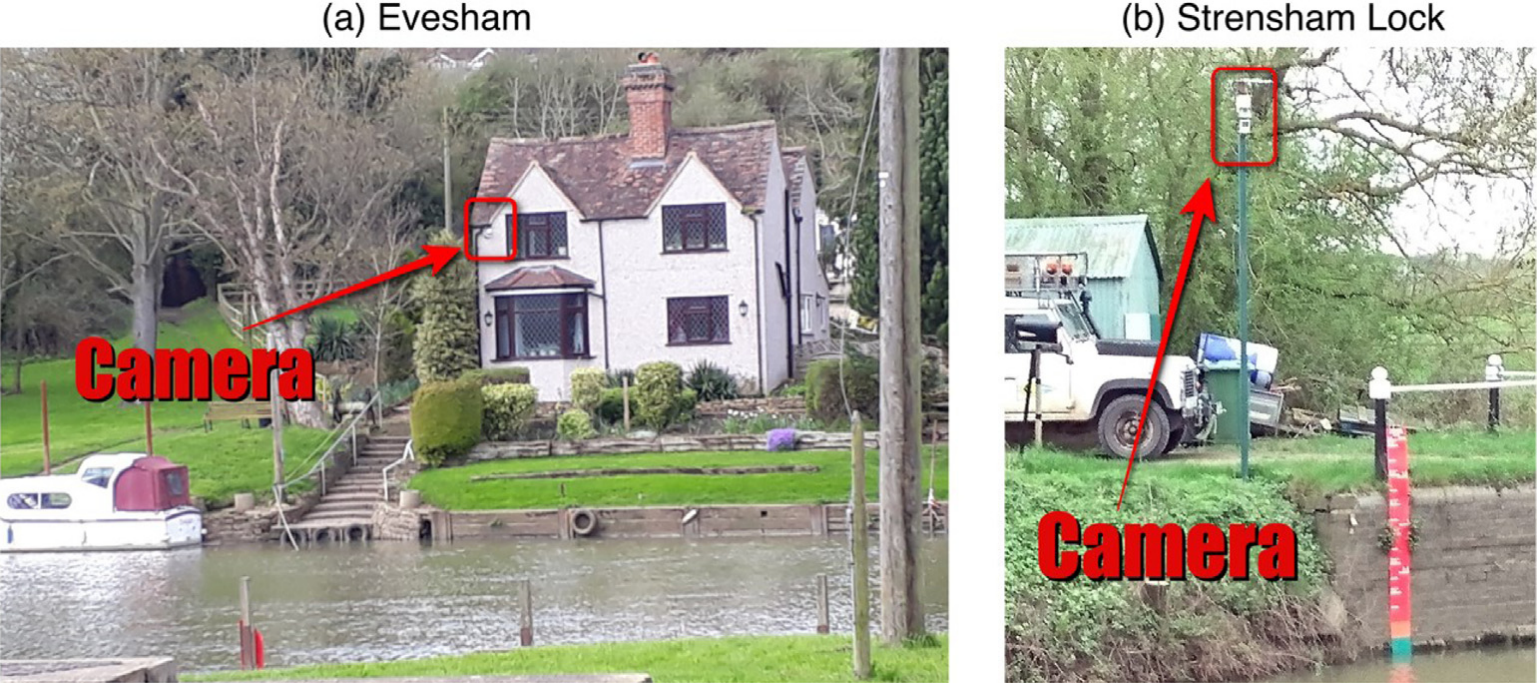
Images of the Farson Digital Ltd river camera installations at (a) Evesham and (b) Strensham Lock.

**Fig. 8:**
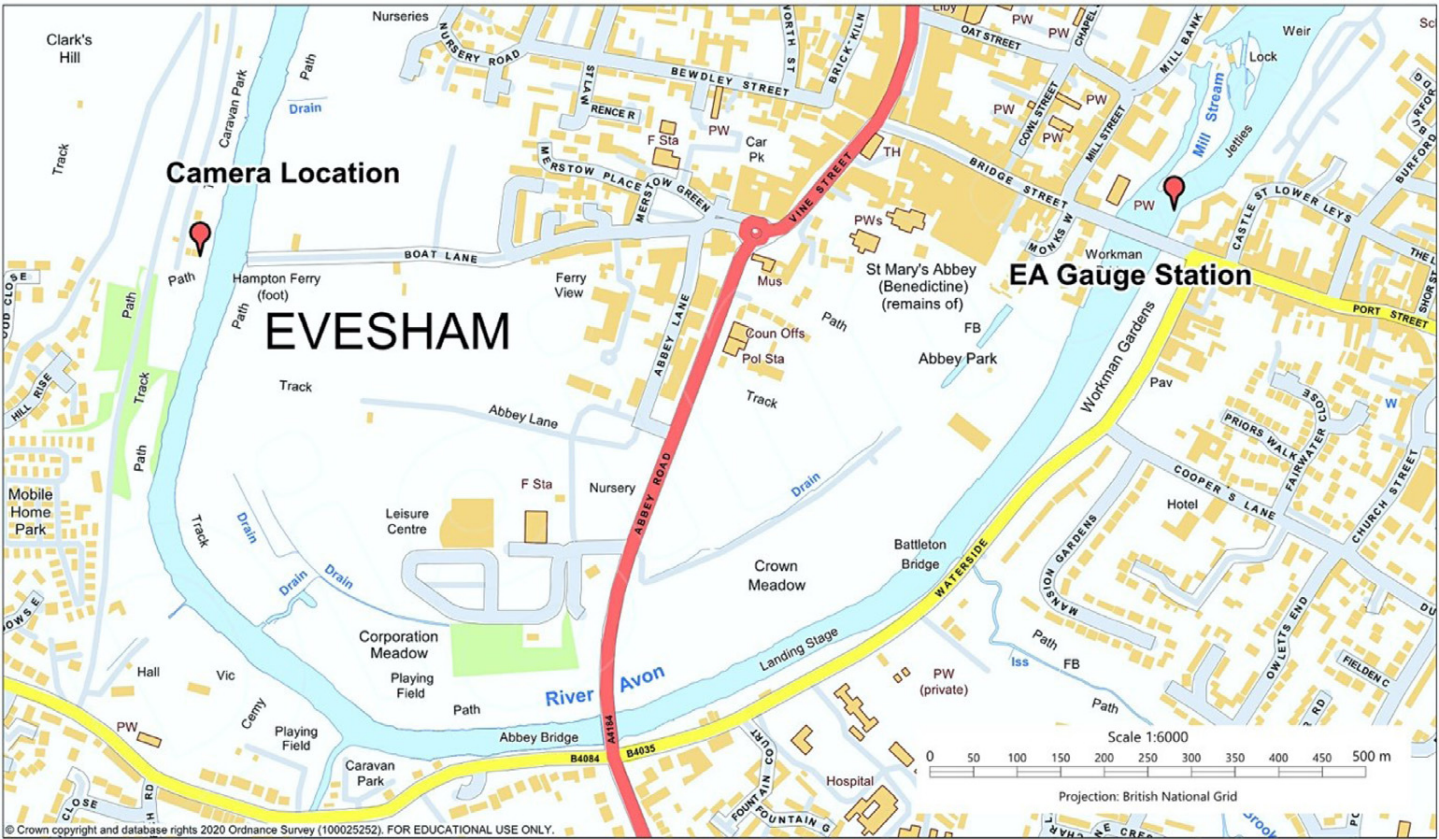
Map illustrating the Evesham camera on river Avon and nearest EA river gauge 'Evesham' (labelled red teardrop shape markers). The map projection is British National Grid and the map scale is given in the bottom right. The vertical is aligned with true North. Produced using EDINA Digimap Ordnance Survey Service, Date created: 09/03/2020. (For interpretation of the references to colour in this figure legend, the reader is referred to the web version of this article.)

**Fig. 9:**
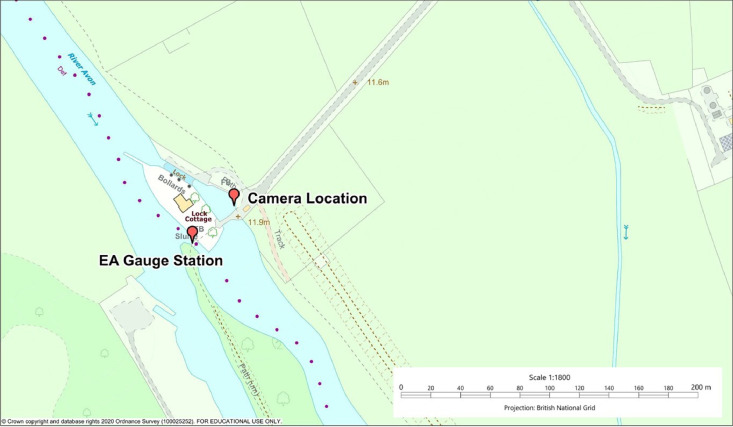
Map illustrating Strensham Lock camera on river Avon and nearest EA river gauge 'Eckington Sluice' (labelled red teardrop shape markers). The map projection is British National Grid and the map scale is given in the bottom right. The vertical is aligned with true North. Produced using EDINA Digimap Ordnance Survey Service, Date created: 09/03/2020. (For interpretation of the references to colour in this figure legend, the reader is referred to the web version of this article.)

**Fig. 10:**
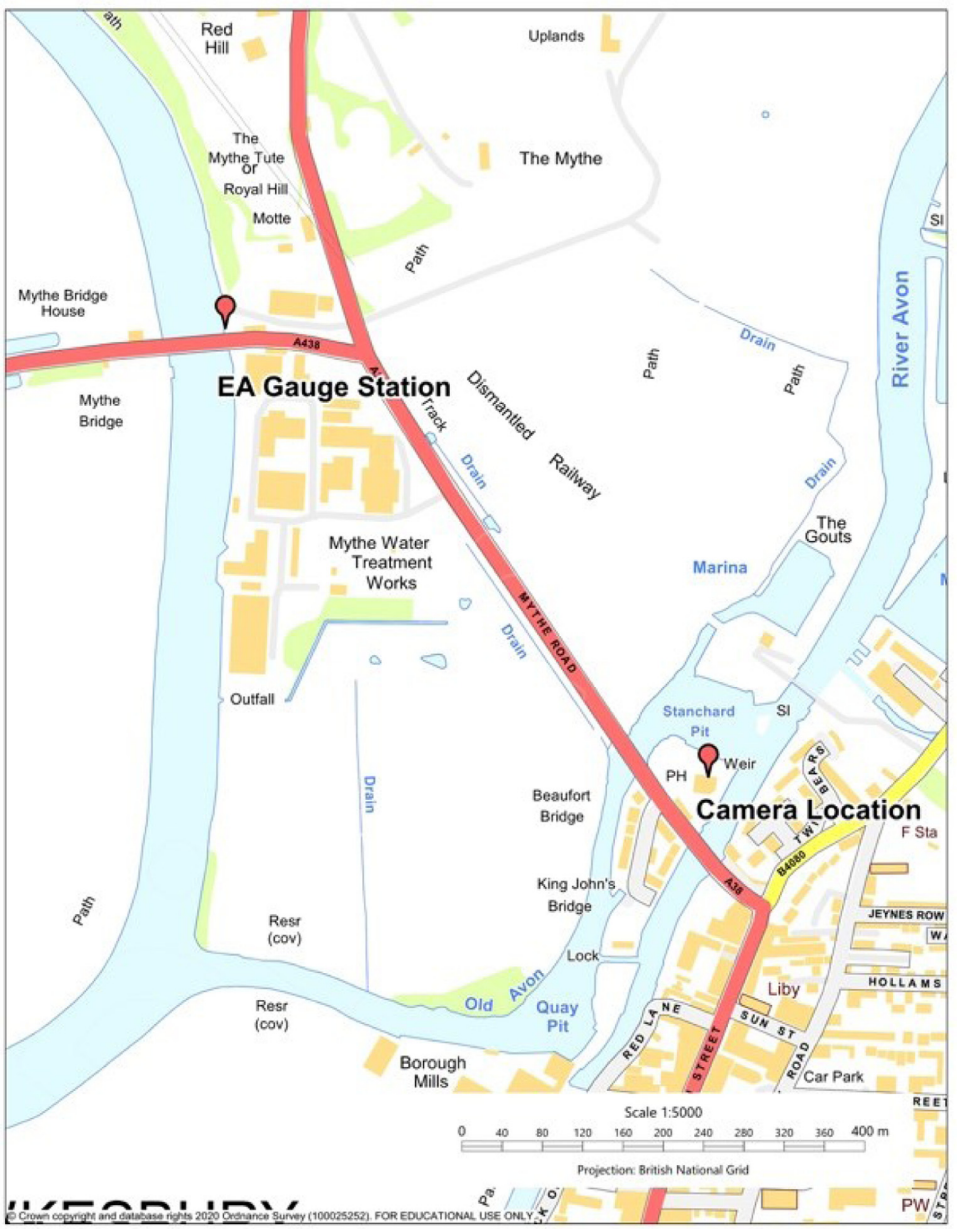
Map illustrating Tewkesbury camera on river Avon and nearest EA river gauge 'Mythe Bridge’ (labelled red teardrop shape markers). The map projection is British National Grid and the map scale is given in the bottom right. The vertical is aligned with true North. Produced using EDINA Digimap Ordnance Survey Service, Date created: 09/03/2020. (For interpretation of the references to colour in this figure legend, the reader is referred to the web version of this article.)

**Fig. 11:**
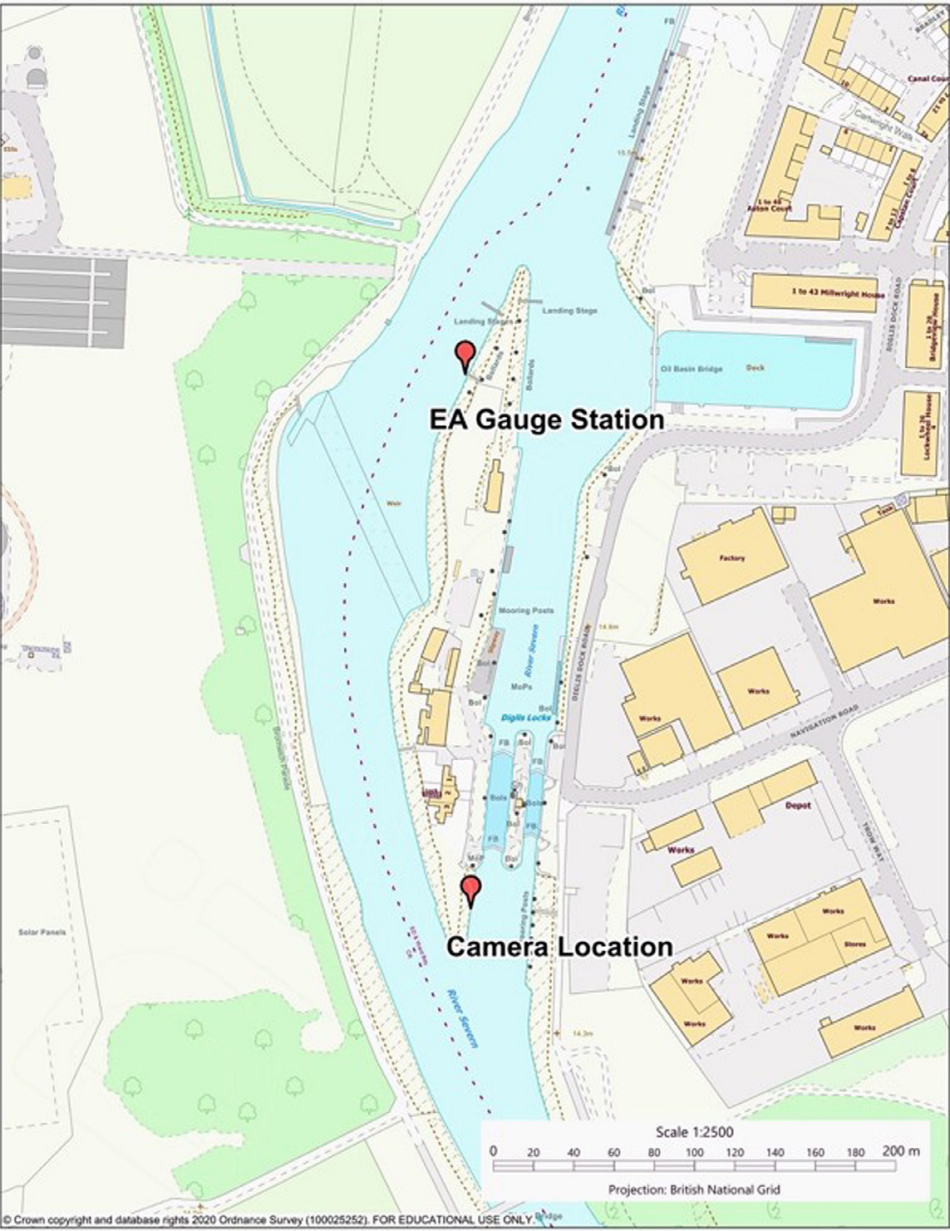
Map illustrating Diglis Lock river camera on river Severn and nearest EA river gauge 'Diglis' (labelled red teardrop shape markers). The map projection is British National Grid and the map scale is given in the bottom right. The vertical is aligned with true North. Produced using EDINA Digimap Ordnance Survey Service, Date created: 09/03/2020. (For interpretation of the references to colour in this figure legend, the reader is referred to the web version of this article.)

**Fig. 12:**
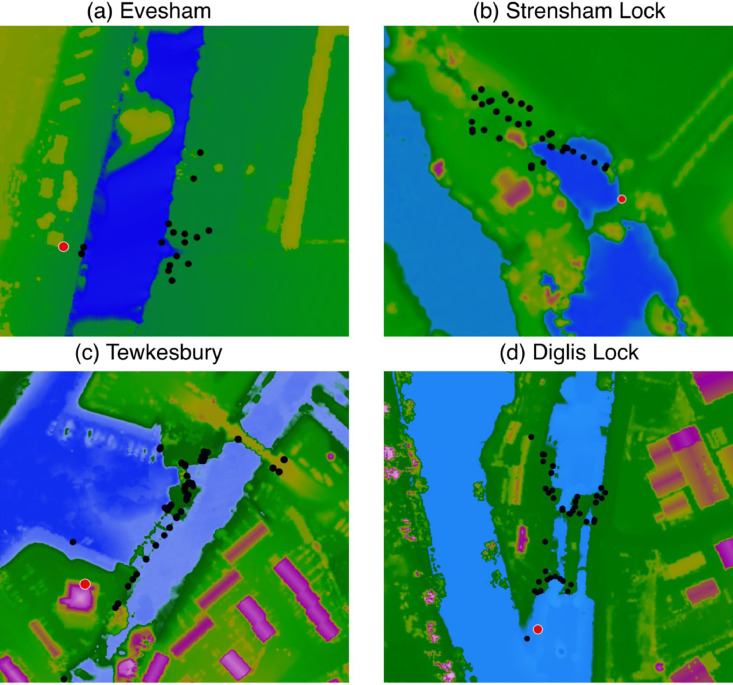
Locations of metadata points for the four river cameras using both the Total Station and the GNSS instruments. The red dots are the camera locations and black dots are the various field-of-view points seen through the cameras. Point data is plotted on the Environment Agency lidar data of 1 m resolution (lidar data can be found here: https://environment.data.gov.uk/DefraDataDownload/?Mode=survey). Use the following references for each of the cameras: sp04sw for Evesham, so94sw for Strensham Lock, so83se for Tewkesbury, and so85sw for Diglis Lock. Note that the colourbar is different for each of the images and since it is irrelevant is not displayed here. Plots made using Landserf Version 2.3. http://www.landserf.org[12]. (For interpretation of the references to colour in this figure legend, the reader is referred to the web version of this article.)

Fig. 1 is a map showing the locations of the four river cameras from which the new water level data is extracted. In Fig. 2, Fig. 3, Fig. 4, Fig. 5 the derived water level data for each of the four cameras (Evesham, Strensham Lock, Tewkesbury and Diglis Lock respectively) is plotted against available river gauge data. The field-of-view of each camera is illustrated in Fig. 6, with yellow dots marking some of the measured metadata locations. The camera installations at two of the sites (Evesham and Strensham Lock) are shown in Fig. 7. The installations at Diglis Lock and Tewkesbury are not shown since the camera locations were moved between the flood event in 2012 and the date of our ground survey. The location of each camera and the nearest EA river gauge are shown in Figs. 8–11 in Appendix A. The measured metadata locations in the field-of-view of each camera are plotted in Fig. 12 in Appendix B.

The analysed water level datasets are provided in data files:

• RiverCamera_WaterLevelObservations_NovDec2012_DiglisLockCamera.xls
• RiverCamera_WaterLevelObservations_NovDec2012_EveshamLockCamera.xls
• RiverCamera_WaterLevelObservations_NovDec2012_StrenshamLockCamera.xls
• RiverCamera_WaterLevelObservations_NovDec2012_TewkesburyLockCamera.xls

available at Mendeley Data repository DOI: 10.17632/769cyvdznp.1. Additionally, the repository also contains the raw unprocessed image data and raw measured location metadata used to produce the analysed datasets.

## 2 Experimental Design, Materials and Methods

### 2.1 Farson digital river cameras

Farson Digital Watercams (https://www.farsondigitalwatercams.com/) is the UK's first network of HD cameras (currently around 155) continuously broadcasting live images from the UK's and Republic of Ireland's waterways, many at ungauged locations. The current network of river camera images is used by a variety of groups including fishermen, tourists, boaters, flood groups, local councils, the Canal & River Trust, and the Environment Agency in England. All the cameras are mounted on buildings, bridges, trees, poles, etc. near rivers at various heights and have a river body in the field-of-view. Thus, river cameras have a high chance of withstanding floods. They are reliant on the availability of electricity, and many carry back-up batteries so that they are still able to function even when the main power supply is disrupted.

The images from the four locations in this study (see Fig. 1) were captured between 21st November 2012 and 5th December 2012, using a Mobotix M24 all-purpose high-definition (HD) web-camera system with 3MP (megapixels) producing 2048 × 1536-pixel images. These cameras were set up to be stationary, i.e. they do not move, tilt, or zoom.

### 2.2 River camera metadata measurement procedure

To extract water level observations (WLOs, shown in Figs. 2–5), a number of reference points on the flood plain in the field-of-view of each river camera are selected. The water level height in the images is assessed against these points. Fig. 6 shows some of the measured points in the field-of-view of each camera marked in the camera image and Fig. 12 in Appendix B shows these locations in plan-view. To ensure that the extracted WLOs are as continuous as possible in time, the points are selected to cover as wide a range of vertical heights as possible. However, due to the specifics of each river camera site there are times at which WLOs are excluded due to a lack of measured reference points at the water level in the image. This happened mainly if all the reference points were flooded (underwater) or when the river was in-bank (on the rising or falling limb) since it was not possible to measure reference points on the riverbanks.

Point coordinates were measured in 3D space using two Leica tools: a GNSS (Global Navigation Satellite System) tool to measure 3D coordinates on any ground point and Total Station to measure any 3D point that was above the ground or inaccessible with the GNSS. Each of these tools produce measurements with uncertainty of O(1 cm) (on order of a centimetre). The two instruments use different versions of the OSGB36 coordinate systems as can be seen in Tables 1 and 3 with GNSS using version OSGB36(15), released in 2016, while Total Station uses the earlier OSGB36(02) system. The shifts in coordinates between the two systems are on average of about 1 cm in the horizontal and approximately 2.5 cm in the vertical across the majority of the UK [11]. Furthermore, the positions of reference stations have changed between two systems. Thus, care needs to be taken to plot the point using the correct coordinate system.

### 2.3 GNSS specifications

To measure 3D coordinates of a ground point a GNSS instrument was used. This connects to a 3 G network to correct itself against a known base-station, producing measurements with accuracy order of 1 cm. The instrument used was a Leica CS10/CS15 & GS Sensor manufactured by Leica Geosystems AG and owned by the University of Reading. The details of the coordinate system used to take the field measurements with the GNSS instrument are given in Table 1 and the base stations are given in Table 2. The GNSS instrument was used to measure the coordinates of the two reference points necessary to set up the Total Station instrument, as described in the next section, as well as to take measurements of ground-based points.

**Table 1:** Details of the GNSS coordinate system used at all of the river camera locations.

Attribute	Description
Coordinate system	OSGB36(15)
Horizontal datum	Local
Vertical datum	Local
Ellipsoid name	GRS 1980
Projected coordinate system name	UKTM

**Table 2:** Details of the 3 G network base stations used at each river camera location to ensure high accuracy GNSS measurements. Note that the WGS stands for World Geodetic System and 3D coordinates in this table have been given based on the details in Table 1.

Base station name	Latitude (degrees, WGS)	Longitude (degrees, WGS)	Height (m, WGS)	Cameras
RTCM-Ref 0179	52.251813367141	−2.197940416267	87.076941	Diglis Lock, Evesham, Strensham Lock, Tewkesbury
RTCM-Ref 0227	51.739170966802	−2.300777891227	76.164304	Tewkesbury

### 2.4 Total station specifications

To measure the 3D coordinates of a point we used a Leica TS12 Lite tool as it allows points above the ground to be measured using either a laser or a prism. The Leica TS12 tool is also manufactured by Leica Geosystems AG and is owned by the University of Reading.

As with the GNSS, the Total Station uses a number of satellites to calculate the 3D position of a point in space. However, the Total Station instrument has to be calibrated against its own backstation prism. For this we used the GNSS instrument to measure the 3D coordinates of both the Total Station instrument and the backstation prism.

The Total Station instrument was used to measure the 3D coordinates of various items in the field-of-view of the camera, such as the top and bottom of buildings, fences, poles, etc. as well as of the river cameras themselves. The details of the coordinate system used to take the field measurements with the Total Station are given in Table 3.

**Table 3:** Details of the Total Station coordinate system used at all of the river camera locations.

Attribute	Description
Coordinate system	OSGB36(02)
Horizontal datum	Local
Vertical datum	Local
Ellipsoid name	GRS 1980
Projected coordinate system name	UKTM

### 2.5 Description of river camera locations

Here we describe the metadata for the river cameras at Evesham, Strensham Lock, Tewkesbury, and Diglis Lock. The physical locations of all the river cameras are shown on the map in Fig. 1 and their 3D coordinates measured using Total Station are given in Table 4. Fig. 7 demonstrates each camera's field-of-view and Fig. 8 illustrates the installations at Evesham and Strensham Lock. The camera installations from Tewkesbury and Diglis Lock are not illustrated since the cameras have moved since the case study dates in 2012.

**Table 4:** 3D coordinates of all river camera positions.

Camera name	Northing	Easting	Height	River
Evesham	243656.208940 m	402923.198359 m	27.581480 m	Avon
Strensham Lock	240449.130905 m	391564.368379 m	15.519703 m	Avon
Tewkesbury	233394.435101 m	389466.950421 m	16.079688 m	Avon
Diglis Lock	253402.082989 m	384691.150601 m	17.777358 m	Severn

We have compared the extracted water level observations to nearby EA river gauge stations. The comparisons are only loose because river gauges provide point measurements of water level that are only strictly valid at the point in the river at which the gauge is located. Additionally, there are a number of weirs and sluices on the rivers that change the behaviour of the river and its water levels. Hence, the WLOs obtained from the river cameras would not necessarily be the same as those obtained from the river gauges. Table 5 gives details of the nearest EA river gauges to each of the river cameras. Their locations are shown on maps in Figs. 8–11 in Appendix A.

**Table 5:** Nearest Environment Agency river gauge stations to each of the river camera locations. The distance given is an approximate distance from the river camera to the nearest EA river gauge along the river network.

Camera name	Gauge name	Gauge location (N/E)	Distance (m)
Evesham	Evesham	243712.5/404037.5	1823 m
Strensham Lock	Eckington Sluice	240418.0/391533.0	51 m
Tewkesbury	Mythe Bridge	233737.5/388887.5	1491 m
Diglis Lock	Diglis	253537.5/384687.5	274 m

### 2.6 Evesham on Avon

The Farson Digital Ltd river camera at Evesham was installed on 12/10/2010 and has had the same view since the initial installation. The camera is mounted on a building (see Fig. 7). The camera field-of-view is seen in Fig. 6 and shows the following large features: the river Avon, the Hampton Ferry boat, Raphael's restaurant, road (Boat lane), and a car park. We measured the 3D coordinates of a selection of points in the camera field-of-view as shown in Figs. 6 and 12.

### 2.7 Strensham Lock on Avon

The Farson Digital Ltd river camera at Strensham Lock was installed on 10/03/2011 and has had the same view since the initial installation. The camera is erected on a pole at the end of the path next to the river (see Fig. 7). The camera field-of-view seen in Fig. 6 has the following large features: a lock on the river Severn, a path with a railing on the right hand side, two large trees, a small green shed with white doors, a wooden fence to the right of the shed, and a number of small poles along the edge of the river. This was the most measured camera location as it was also used as a demonstration site for visiting colleagues. Hence, there is a much denser and larger number of measurements as seen in Figs. 6 and 12.

### 2.8 Tewkesbury on Avon

The Farson Digital Ltd river camera at Tewkesbury was originally installed on 10/03/2011 and until 2016 was located on the wall of a building. This was the position of the camera at the time of taking images for the 2012 Tewkesbury flood event; however, since 2016, the camera has been relocated on to the bridge close by. The original location of the camera is shown in Fig. 10. The camera field-of-view seen in Fig. 6 has the following large features: a grass patch, boats, a weir, and the river Avon. We measured the 3D coordinates of a selection of points in the camera field-of-view seen in Fig. 12.

### 2.9 Diglis Lock on Severn

The Farson Digital Ltd river camera at Diglis Lock was originally installed on 20/04/2012 and until 24/11/2016 was located on a signpost. This was the position of the camera at the time of the 2012 Tewkesbury flood event; however, since 2016, the camera has been relocated on to the Diglis Cottage building close by. The original location of the camera is shown in Fig. 11. The camera field-of-view seen in Fig. 6 has the following large features: a grass patch, two large metal poles, the lock, and the River Severn. We measured the 3D coordinates of a selection of points in the camera field-of-view as shown in Fig. 12.

### 2.10 Extraction of water-level information

Each individual image was manually assessed to determine the water height based on known landmark locations once the river was out of bank. For each image the measured landmarks were marked flooded or unflooded and the water level estimated based on the lower bound (the height of the highest flooded landmark) and the upper bound (the height of the lowest unflooded landmark).

## CRediT Author Statement

**Sanita Vetra-Carvalho:** Conceptualization, Methodology, Validation, Investigation, Data Curation, Writing - Original Draft, Visualization, Supervision, Project administration. **Sarah L. Dance:** Conceptualization, Methodology, Investigation, Writing - Review & Editing, Supervision, Project administration, Funding acquisition. **David C. Mason:** Conceptualization, Methodology, Investigation, Writing - Review & Editing, Supervision, Funding acquisition. **Joanne A. Waller:** Investigation, Writing - Review & Editing, Supervision. **Elizabeth S. Cooper:** Investigation, Writing - Review & Editing. **Polly J. Smith:** Investigation, Writing - Review & Editing. **Jemima M. Tabeart**: Investigation, Writing - Review & Editing

## Declaration of Competing Interest

This work was funded by the Data Assimilation for the REsilient city (DARE) project, an EPSRC Senior Fellowship in Digital Technology for Living with Environmental Change (EPSRC EP/P002331/1). River camera data were obtained at no cost from Farson Digital Ltd. The authors declare that they have no other known competing financial interests or personal relationships which have, or could be perceived to have, influenced the work reported in this article.
